# Geographic authentication of premium tobacco extracts using machine learning models trained on LC-HRMS fingerprints

**DOI:** 10.3389/fmolb.2026.1898152

**Published:** 2026-08-27

**Authors:** Xiu-Juan Xu, Min Wang, Xiao-Ping Li, Wei-Chen Zhang, Shi-Tong Zeng, Qing-Zhao Shi, Yi-Wei Ma, Bin-Bin Lu, Shan Liu, Ding-Zhong Wang, Ren-Qi Wang, Jun Hu

**Affiliations:** 1 Zhengzhou Tobacco Research Institute of China National Tobacco Corporation, Zhengzhou, China; 2 School of Food and Biological Engineering, Shaanxi University of Science and Technology, Xi’an, China; 3 Jiangsu Xinyuan Reconstituted Tobacco Co., Ltd., Huai’an, China

**Keywords:** authentication, fingerprinting, LC-MS, machine learning, nontargeted analysis

## Abstract

**Introduction:**

The geographic origin of premium tobacco extracts fundamentally shapes their sensory profiles and commercial value, but authenticating these complex matrices remains a formidable analytical challenge. Conventional nontargeted analysis workflows typically rely on a minute fraction of structurally annotated metabolites, thereby discarding the vast majority of unannotated yet highly specific chemical markers. To address this limitation, this study introduces a geographic authentication framework utilizing machine learning models trained on characteristic ion feature (CIF) fingerprints.

**Methods:**

A large-scale dataset of 1,697 tobacco samples was analyzed using liquid chromatography-high resolution mass spectrometry coupled with a data-independent acquisition strategy. Rigorous statistical filtering was applied to isolate CIFs and strip away the shared background metabolome. To evaluate the samples, univariate thresholding based on pattern similarity (dot product) and marker intensity ratios (R score) was initially tested. To overcome the vulnerability of univariate methods to batch effects and overlapping chemical profiles, optimized multivariate machine learning models were also deployed. The models were evaluated under a strict Leave-One-Batch-Out cross-validation framework to ensure robust cross-batch generalization.

**Results:**

Statistical filtering effectively stripped away over 90% of the shared background metabolome, isolating 47,543 CIFs for Zimbabwe tobacco and 3,646 CIFs for Yunnan tobacco. Univariate thresholding achieved a maximum balanced accuracy of 77.9% but proved too rigid. Under cross-validation, the multivariate Random Forest (RF) classifier demonstrated strong predictive performance, achieving a balanced accuracy of 74.6% for Zimbabwe tobacco and successfully detecting economically motivated adulteration across a wide dilution range (0–1,000 ppm). Conversely, both univariate and multivariate methods failed to achieve robust authentication for the Yunnan extracts. This underperformance was driven by severe intra-group heterogeneity stemming from diverse suppliers and heterogeneous processing treatments, which heavily diluted universal characteristic features.

**Discussion:**

Ultimately, this hybrid strategy provides a scalable tool for the industrial verification of consistent premium extracts. Furthermore, the challenges encountered with the Yunnan samples underscore the necessity of cohort-specific modeling when dealing with highly diverse agricultural products that are subjected to varied processing treatments.

## 1 Introduction

The geographic origin fundamentally shapes the metabolic composition of tobacco, which in turn determines its sensory characteristics and commercial value for cigarette manufacturing. Driven by highly favorable growing environments featured by intense ultraviolet radiation and distinct day-night temperature shifts, flue-cured tobaccos from Zimbabwe and the Yunnan province of China are greatly prized for their premium flavor profiles and extensively used as high-value flavor-enhancing materials (Li et al., 2011; Wu et al., 2026). Consequently, the reliable authentication of their geographic origins is of considerable industrial importance. However, authentication becomes exceptionally challenging once raw tobacco leaves are processed into extracts that evade standard morphological inspection. Although sensory evaluation by expert panels has traditionally been employed as an authentication strategy, this approach is inherently subjective, low throughput, and prohibitively expensive for large-scale industrial screening.

To address these analytical challenges, nontargeted analysis (NTA) based on gas chromatography-mass spectrometry (GC-MS) or ultrahigh performance liquid chromatography-high resolution mass spectrometry (UPLC-HRMS) has emerged as a powerful analytical strategy for profiling complex extracts. Currently, the dominant research paradigm relies on multivariate statistics, such as Principal Component Analysis (PCA), Orthogonal Partial Least Squares Discriminant Analysis (OPLS-DA) and Hierarchical Cluster Analysis (HCA), to isolate and structurally annotate differential metabolites acting as discriminatory markers for geographic classification (Li et al., 2011; Zhang et al., 2013; Tie et al., 2024; Xu et al., 2025). While these descriptive profiling approaches have successfully mapped regional flavor differences to their underlying biosynthetic pathways, they are fundamentally limited by the reliance on annotation (Liu et al., 2022; Chen et al., 2025; Zhao et al., 2026). Due to the constraints of current MS databases and annotation algorithms, it is estimated that less than 2% of features in complex natural extracts can be successfully annotated (da Silva et al., 2015). Consequently, classification methods that strictly discard the vast majority of unannotated metabolites risk overlooking highly discriminatory markers, thereby potentially constraining the predictive robustness and overall accuracy.

To overcome the limitations of annotation-dependent workflows, holistic metabolite fingerprinting serves as a robust alternative. Rather than identifying individual compounds, this method leverages the entire chromatographic or spectral profile as a comprehensive molecular signature (Riswanto et al., 2022). The predictive capability of fingerprinting has been fundamentally transformed by the integration of advanced machine learning (ML) algorithms capable of resolving high-dimensional data matrices. Supervised classification models have vastly outperformed traditional unsupervised methods like PCA in tracing agricultural origins (Ratnasekhar et al., 2025; Wu et al., 2026). Because the metabolic differences between geographical regions are often highly entangled, non-linear algorithms such as Support Vector Machine (SVM) and Random Forest (RF) are recognized as highly suitable (Li et al., 2024). Therein, hybrid-kernel SVMs address the complexity of mass spectral fingerprints by mapping data into higher-dimensional spaces to find clear separation boundaries, while optimized RF models navigate entanglement by recursively partitioning the feature space through dense decision trees (Lin et al., 2010; Dawei et al., 2025; Ratnasekhar et al., 2025; Kou et al., 2026).

Despite these algorithmic advancements, the sheer dimensionality and chemical redundancy inherent in raw holistic fingerprinting datasets persistently present a substantial challenge (Picone, 2026). Unfiltered profiles encompass irrelevant background noise and shared baseline metabolites that actively dilute the discriminatory power of even the most sophisticated ML models. To overcome this, an emerging hybrid strategy refines NTA fingerprints through multivariate analysis prior to training ML models (Gromski et al., 2014; Saha et al., 2026). Rather than feeding an unfiltered dataset into an algorithm, or conversely, limiting the data strictly to structurally annotated metabolites, this combined approach mathematically extracts unannotated but significantly differential ion features to construct the ML models. By filtering the holistic fingerprint down to its most discriminative components prior to classification, this strategy merges targeted precision with annotation-independent ML, maximizing predictive accuracy and computational efficiency.

While hybrid classification strategies show immense promise, their application is fundamentally constrained by a critical technical bottleneck: reliably tracking the same unannotated ion feature across different analytical batches. In large-scale chromatographic analyses, inevitable variations such as retention time drift make the cross-sample alignment of unannotated features highly challenging (Lange et al., 2008). Because these features lack structural identities to act as definitive reference points, tracking them across multiple sample batches becomes highly susceptible to misalignment. Consequently, this persistent alignment barrier has hindered the development of authentication models trained by large-scale datasets attained from samples across different batches. Without these models, it is incredibly difficult to distinguish premium agricultural products from lower-quality alternatives since they share highly similar chemical matrices.

To bridge this gap, we recently developed a biogenic solution mapping algorithm. This approach integrates a standardized LC-HRMS NTA protocol with a tandem mass spectrometry-based peak alignment algorithm, enabling the efficient matching of identical ion features across different analytical batches (Wang et al., 2023). By applying statistical feature selection, we extracted tens of thousands of highly discriminative ion features to construct robust Characteristic Ion Feature (CIF) matrices. This strategy successfully distinguishes Zimbabwe and Yunnan tobaccos while circumventing the traditional bottleneck of structural annotation. Leveraging a large-scale dataset of 1,697 samples from 14 analytical batches, which specifically includes 163 authentic Zimbabwe and 440 Yunnan tobacco extracts, we developed a composite scoring algorithm that incorporates dot product similarity and intensity ratios. Finally, we trained and evaluated RF and hybrid-kernel SVM classifiers to establish an optimized authentication threshold. By transitioning from descriptive metabolite profiling to predictive, high-dimensional ML models, this work aims to provide a robust, scalable, and specific tool for the rapid industrial identification of premium tobacco materials.

## 2 Materials and methods

### 2.1 Sample preparation

A total of 1,697 plant extracts were purchased from local markets in mainland China, comprising 1,454 tobacco extracts and 243 extracts from other plants. Detailed information regarding the extracts is provided in Data Sheet 1 (Supporting Data).

For validation purposes, Zimbabwe tobacco extracts were prepared in the laboratory. Briefly, 30 g of tobacco leaves were crushed into a powder, transferred to a round-bottom flask, and mixed with 240 g of 70% (v/v) ethanol. The mixture was stirred and heated at 75 °C for 3 h, then filtered through a cloth to collect the primary filtrate. The remaining filter residue was re-extracted with 180 g of 70% (v/v) ethanol at 75 °C for 2 h and filtered through a 300-mesh cloth. The two filtrates were combined and subjected to secondary filtration through a funnel to yield a tincture. Finally, the tincture was concentrated under vacuum using a rotary evaporator (water bath at 45 °C, cooling bath at 4 °C, vacuum at 0.1 bar) to an ointment-like consistency to yield the final extract. Non-Zimbabwe tobacco extracts were prepared using tobacco leaves from blank cigarettes following the same extraction protocol. These non-Zimbabwe extracts were subsequently spiked with the aforementioned Zimbabwe extracts (aged in the laboratory for 2 weeks) to achieve concentrations of 0, 10, 100, and 1,000 ppm. For NTA, 1 mL of each prepared extract was mixed with 4 mL of methanol, centrifuged at 10,823 × *g* for 10 min at 4 °C, and the resulting supernatant was transferred to HPLC vials for subsequent analysis.

### 2.2 LC-HRMS analysis and data preprocessing

Liquid chromatography–high resolution mass spectrometry (LC–HRMS) analysis was carried out using a Waters UPLC system coupled with a Sciex 5600 TOF MS instrument. Chromatographic separation was conducted using both reversed-phase liquid chromatography (RPLC) and hydrophilic interaction liquid chromatography (HILIC) to enhance metabolite coverage across a broad polarity range. RPLC separation was performed on a Waters ACQUITY UPLC HSS T3 column (130 Å, 1.7 µm, 2.1 × 100 mm), whereas HILIC separation was carried out on a Waters ACQUITY UPLC BEH Amide column (130 Å, 1.7 µm, 2.1 × 100 mm). Both chromatographic modes employed a binary mobile phase system consisting of water and acetonitrile, with 0.1% formic acid added to both phases. The flow rate was set at 0.3 mL/min, and the column temperature was maintained at 40 °C. Quality control (QC) samples were prepared by pooling equal volumes of all samples and analyzed using a standardized nine-gradient elution program (Wang et al., 2023).

Data acquisition was performed using a data-independent acquisition (DIA) strategy in both positive and negative ionization modes (Wang et al., 2023). TOF MS full-scan data were acquired over an m/z range of 70–1,200 Da with an accumulation time of 150 m. The DIA method employed 12 variable-width SWATH windows, with an MS/MS scan time of 50 m for each window. The declustering potential (DP), collision energy (CE), and collision energy spread (CES) were set to 80 V, 35 V, and 15 V, respectively. Comprehensive data processing and nontargeted metabolite annotation were conducted using the integrated chromatographic retention behavior–fragmentation chain characterization (CRB-FCC) workflow (Wang et al., 2021; Wang et al., 2025). Based on the nontargeted ion feature list extracted from the QC dataset using the CRB algorithm, targeted searches were performed for corresponding nontargeted ion peaks in the LC-MS/MS data of each designated sample.

Unlike conventional nontargeted metabolomics workflows that rely on study-specific pooled quality control (QC) samples for signal drift correction, thereby limiting long-term inter-batch comparability, our workflow employs intrinsic physicochemical properties of molecular features for cross-batch alignment without external QC calibration. Peak extraction, retention time alignment, and feature matching were conducted using a precursor mass tolerance of 10 ppm and 20 ppm in positive and negative ion modes. Retention time alignment across analytical batches was performed using a maximum drift tolerance of 1 min to accommodate chromatographic variation while minimizing false-positive feature matching. Tandem mass spectra were subsequently matched based on fragmentation-chain similarity, with feature pairing requiring a minimum positive matching rate of 50%. Data acquired under RPLC positive-ion, RPLC negative-ion, HILIC positive-ion, and HILIC negative-ion conditions were processed independently to generate mode-specific feature matrices. Finally, redundant features detected across multiple chromatographic modes or ionization polarities were consolidated by retaining the feature exhibiting the highest peak area for downstream statistical analysis.

### 2.3 Characteristic ion feature (CIF) extraction

Data processing and statistical analyses were conducted on the fingerprint data matrices of 1,697 samples, which included 163 Zimbabwe tobacco extracts and 440 Yunnan tobacco extracts. To identify differential ion features exhibiting significantly higher abundance in these specific sample groups, Welch’s t-test was used to compare each target group against a pooled background of all remaining samples. Specifically, the mean intensity of each ion feature within the Zimbabwe cohort (n = 163) was compared against the mean intensity of the remaining non-Zimbabwe samples (n = 1,534). An analogous comparison was performed for the Yunnan cohort (n = 440) against all non-Yunnan samples (n = 1,257). Characteristic ion features (CIFs) for the Zimbabwe and Yunnan groups were selected based on the following stringent criteria: fold change > 2, p < 0.05, q < 0.01, and a mean intensity exceeding 1,000 cps in the target group.

### 2.4 Univariate thresholding algorithm

To establish baseline profiles, reference CIF matrices for the Zimbabwe and Yunnan groups were first constructed using the m/z, t_R_, and group-averaged intensities of their respective characteristic features. After that, the corresponding intensity values for these specific CIFs were extracted from the fingerprint data of all 1,697 individual samples to generate sample-specific feature matrices. The dot product (DP) was then calculated between each sample matrix and the two reference CIF matrices, yielding two distinct similarity scores per sample based on our previous report (Wang et al., 2023) (Equations 1, 2).

$${DP}_{r,s}=\frac{{\left(\sum W_{r,i}W_{s,i}\right)}^{2}}{\sum W_{r,i}^{2}\sum W_{s,i}^{2}}$$ (1)

$$W_{r,i}={t_{R}}_{i}^{1}\times {\left(mz\right)}_{i}^{1}\times {\left(\frac{I_{r,i}}{\max\left(I_{r}\right)}\right)}^{0.5}$$ (2)

Here, DP between reference samples *r* and *s* was calculated based on the composite weighted vectors (*W*) that integrate three orthogonal dimensions of each ion feature, i.e., chromatographic retention time (*t*_*R*_), *m/z*, and intensity (*I*) which was normalized by the base-peak (max*(I)*). Incorporating *t*_*R*_ and *m/z* directly into the feature weights ensures that chemical matches are rigorously constrained by both temporal elution and exact mass, rather than relying on spectral abundance alone. The intensity parameter is scaled by an exponential factor of 0.5.

Complementing the qualitative similarity matching provided by the DP value, the summed intensity ratio (*R*, Equation 3) serves as a macroscopic quantitative metric. Rather than evaluating spectral similarity, it represents the overall proportional contribution of the characteristic ion features relative to the entire chemical background of the sample matrix.

$$R=\frac{\sum_{i=1}^{n}I_{CIF,i}}{\sum_{j=1}^{N}I_{Total,j}}$$ (3)

Therein, *R* represents the score of summed intensity ratio for a given sample, yielding a fractional value between 0 and 1 that reflects the proportion of the total detectable mass comprised of the specific chemical markers. This metric is derived by first calculating the numerator, *I*_*CIF,i*_, which is the absolute intensity of the *i-th* characteristic ion feature across *n* total valid features defining the target reference group (e.g., Zimbabwe or Yunnan tobacco). This targeted sum is then normalized against the entire chemical background of this sample, represented in the denominator by *I*_*Total,j*_, which denotes the absolute intensity of the *j-th* ion feature out of *N* total valid features extracted during the NTA fingerprint of that specific sample.

To provide a comprehensive evaluation of how strictly chemical fingerprint of a sample conforms to the characteristic ion feature (CIF) profiles of the target tobacco groups, a composite similarity factor (*Combo*, Equation 4) was derived. Therein, the *DP* value evaluates qualitative structural alignment and the *R* score measures relative quantitative abundance.

Combo=DP×R (4)

Following the computation of the DP, R, and Combo scores for all 1,697 samples, optimal classification thresholds were established to definitively distinguish between authentic and non-authentic tobacco extracts. To determine these critical cut-off values, two statistical approaches were employed. First, the Probability Density Function (PDF) intersection method was utilized to identify the point at which the distribution curves of the authentic and non-authentic cohorts intersect, representing the theoretical threshold that minimizes total classification error (Kittler and Illingworth, 1986). To empirically validate and refine this boundary, Receiver Operating Characteristic (ROC) curve analysis was conducted to calculate Youden’s J statistic (J = Sensitivity + Specificity-1). By determining the point that maximizes Youden’s index, the optimal threshold yielding the highest combined true positive and true negative classification rates was selected for the final authentication framework (Youden, 1950).

### 2.5 Machine learning classification and hyperparameter optimization

To authenticate premium tobacco varieties (i.e., Zimbabwe and Yunnan), classification models were developed using fingerprints of 1,697 samples attained in 14 batches. To robustly evaluate model performance and account for potential batch effects, a Leave-One-Batch-Out (LOBO) cross-validation framework was implemented. To prevent data leakage and mitigate the curse of dimensionality, feature screening was performed strictly on the training folds within each LOBO iteration. Features were filtered using univariate statistical thresholds (Student’s t-test with FDR q < 0.01, Fold Change > 2, and mean intensity > 1,000), with the candidate pool restricted to the top 1,000 most significant features based on PCA importance ranking.

A Random Forest (RF) classification model was trained on the screened features using Bootstrap Aggregation, ensuring tree independence through random feature subsampling at each node split. To dynamically determine the optimal model architecture, Bayesian optimization within a nested 5-fold cross-validation framework was utilized to tune the number of input features, the number of trees, and the minimum leaf size. To address inherent class imbalances and prevent majority-class bias, a custom cost matrix was optimized and integrated during training to heavily penalize false-negative misclassifications.

In parallel, a Support Vector Machine (SVM) algorithm utilizing a Radial Basis Function (RBF) kernel was developed to provide a comparative geometric classification boundary. This model utilized the identical LOBO partitioning and univariate feature screening established in the primary pipeline. Prior to RBF distance calculations, the selected mass spectrometry features were centered and scaled using Z-score standardization. Class imbalance was similarly addressed using an optimized cost matrix. The SVM hyperparameters, i.e., the number of features, cost penalty, box constraint (*C*), and kernel scale (γ^−1^), were dynamically refined using nested 5-fold Bayesian optimization configured for 50 objective evaluations.

The fully optimized RF and SVM models were iteratively evaluated solely on the unseen LOBO holdout batches. Final predictive performance was comprehensively assessed across the entire dataset using overall accuracy, sensitivity, specificity, and balanced accuracy. To evaluate the statistical reliability of the results, 95% confidence intervals (CIs) for all metrics were calculated using a bootstrapping approach with 1,000 iterations, and model significance was confirmed via permutation testing.

## 3 Results and discussion

### 3.1 Construction of characteristic ion feature (CIF) matrices of Zimbabwe and yunnan tobaccos

**FIGURE 1:**
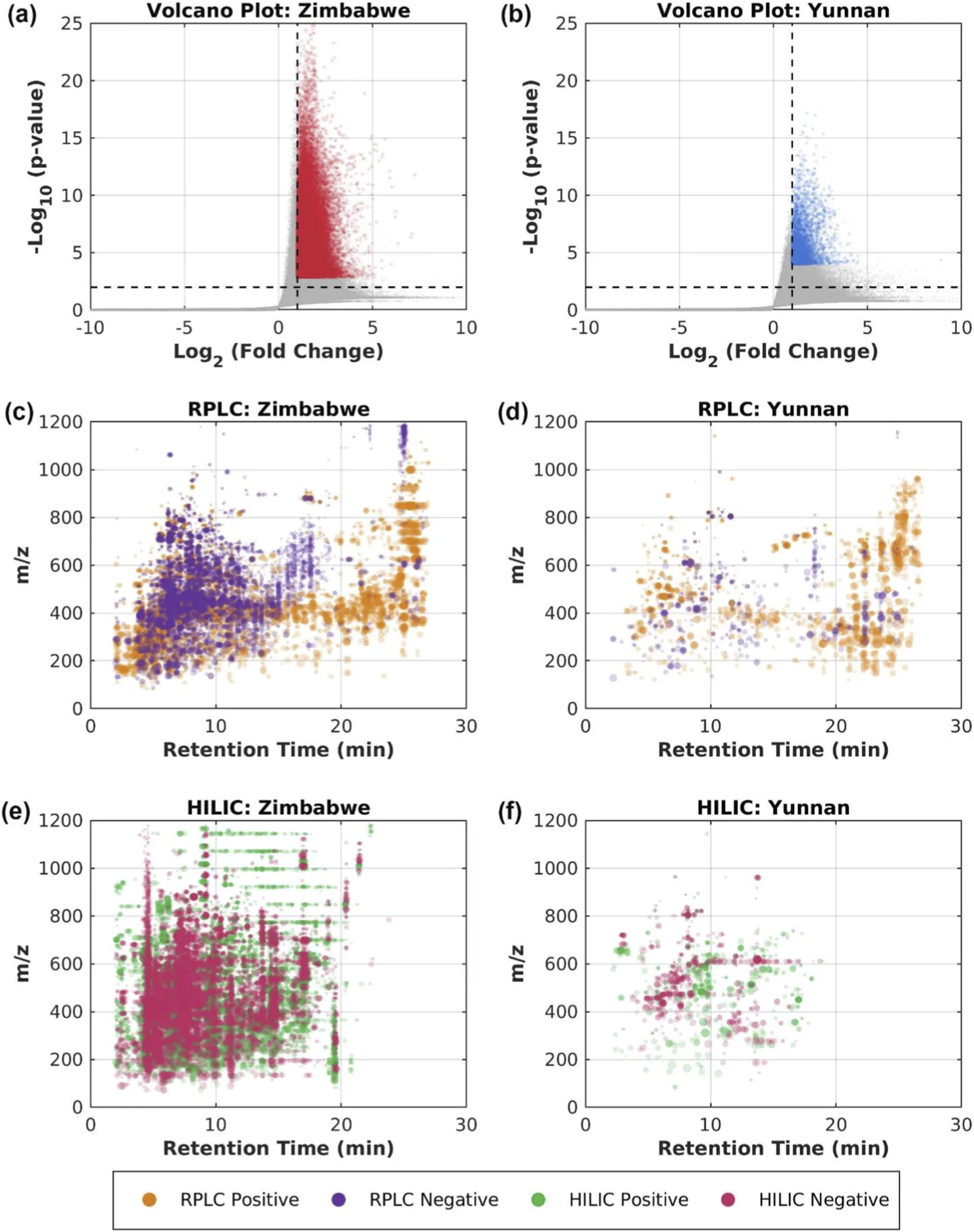
Macroscopic distribution of Characteristic Ion Feature (CIF) matrices from premium tobacco extracts via LC-HRMS nontargeted analysis **(a,b)** Volcano plots for Zimbabwe and Yunnan tobaccos. Significant features (q < 0.01, Fold Change >2, Mean Intensity >1,000) are colored; non-significant features are gray **(c–f)** Cloud plots of significant CIFs detected under RPLC **(c,d)** and HILIC **(e,f)**. Features are mapped by retention time (x-axis) and m/z (y-axis). Bubble size reflects log10 (mean intensity), and colors denote analytical modes.

To map the macroscopic chemical divergence between premium tobacco extracts, 1,697 samples were analyzed by a standardized 9-gradient LC-HRMS protocol coupled with DIA MS technique. Raw spectral data were first processed using the CRB deconvolution algorithm to resolve overlapping ion features. Subsequently, the FCC algorithm was deployed to assign empirical formulas to these signals, and any features without deconvolved MS/MS spectra were strictly discarded (Wang et al., 2021; Wang et al., 2025). To maximize data fidelity and minimize artifacts, signals were retained as authentic molecular features only when consistently assigned identical molecular formulas across samples. This strategy preserved over 90% of detectable compounds while excluding background noise (Wang et al., 2025). Through this rigorous data curation pipeline, a comprehensive global matrix comprising 801,645 validated, reproducible ion features was consolidated across the four complementary analytical modes. From this massive global data pool, strict statistical filtering criteria (i.e., q < 0.01, Fold Change > 2, and Mean Intensity > 1,000) were applied to extract the Characteristic Ion Features (CIFs) for the two premium tobacco types. This analysis yielded 47,543 CIFs for the Zimbabwe group and 3,646 CIFs for the Yunnan group, representing merely 5.9% and 0.4% of the total feature pool (Characteristic Ions, Supporting Data). This highlights the precise discriminatory power of the statistical filtering, which successfully stripped away over 90% of the shared background metabolome to isolate only the most highly specific regional markers. The absolute necessity of this rigorous statistical stringency is visually captured in the volcano plots (Figures 1a,b). While the horizontal dashed line indicates the conventional raw p-value threshold (i.e., p = 0.01, corresponding to a -log_10_(p) of 2), the colored spots representing the true CIFs only appear at significantly higher -log_10_(p) values. This upward shift reflects the strict implementation of the False Discovery Rate (FDR) correction (i.e., q < 0.01). In a dataset containing over 800,000 variables, standard p-values inevitably generate massive quantities of false positives. By enforcing the strict q-value threshold, only features with exceptionally robust, corrected statistical significance were retained, guaranteeing that the differential matrices are built on true geographical differences rather than mathematical artifacts. The stark contrast in CIF quantities reflects fundamental differences in the agricultural and processing consistencies of the two premium groups. The yield of a large number of CIFs for the Zimbabwe group signifies a highly stable metabolic profile and consistent quality across its samples. Conversely, the severely restricted CIF pool for the Yunnan group is indicative of a highly diverse sample cohort. Driven by a broader network of diverse suppliers and heterogeneous processing treatments, the internal chemical complexity of the Yunnan extracts is vastly increased. Consequently, universal characteristic features that define the entire Yunnan cohort are heavily diluted, making a singular, homogenous fingerprint difficult to isolate.

To further elucidate the spatial and physicochemical distribution of these differential markers, cloud plots mapping the features by retention time and m/z value were evaluated (Figures 1c–f). The distribution patterns clearly validate the necessity of the multi-mode chromatographic analysis, as distinct chemical classes were widely partitioned according to their polarity. Notably, the significantly larger number of CIFs demonstrates that Zimbabwe tobacco extracts possess a significantly richer metabolic signature than the comparatively less feature-rich Yunnan tobacco extract. In RPLC mode, this divergence is starkly illustrated in the retention time window of 15–20 min (Figures 1c,d). Although CIFs of both Zimbabwe and Yunnan share a cluster of positive ions in the low mass range (i.e., m/z < 400), Zimbabwe CIFs exhibit a concentrated distribution of negative ions in the high mass range (i.e., m/z 400–600), which correlate strongly with complex, acidic species such as polyphenols and glycosylated flavonoids (Kou et al., 2026; Wu et al., 2026). Furthermore, RPLC-positive mode reveals a heavily retained cluster (t_R_ 20–27 min), capturing highly hydrophobic species critical to tobacco aroma, including complex terpenoids, long-chain fatty acid esters, and lipid-like conjugates (Hu et al., 2025; Wu et al., 2026). On the other hand, the HILIC mode exhibits a massive abundance of Zimbabwe CIFs in the medium polarity range (i.e., t_R_ 4–20 min; m/z 100–800, Figure 1e). In perusal of ions in this range, we can observe series of vertical spot lines, indicating multiple adduct formation from single parent molecules. Meanwhile, series of horizontal spot lines in positive ion mode in retention time window 10–20 min were also observed, which illustrate the successful temporal resolution of isomeric compounds. Yunnan CIFs detected in HILIC mode are also localized within the same spatial window (t_R_ 4–20 min; m/z 100–800, Figure 1f). However, owing to the lower number of Yunnan CIFs, distinct vertical and horizontal spot lines observed in Zimbabwe CIFs fail to emerge.

Despite the quantitative differences in feature density, the overall spatial distribution framework of CIFs across both Zimbabwe and Yunnan tobaccos exhibits underlying similarities. This indicates that despite geographical and phenotypic divergences, the foundational biosynthetic pathways remain conserved, yielding analogous chemical families such as polyphenols, alkaloids, and diterpenoids. The heightened signal intensities observed within specific geometric zones highlight a substantial upregulation and structural diversification of these established pathways, likely a physiological adaptation to regional environmental stressors such as elevated altitude and UV exposure. Because these structural analogues and metabolic derivatives share core skeletal frameworks, their overlapping polarities and molecular weights dictate their tight clustering. This observation aligns with established literature characterizing the retention behavior of plant metabolite homologous series, demonstrating that environmental pressures drive quantitative enrichment and minor enzymatic modifications rather than the genesis of entirely novel biochemical pathways (Wu et al., 2026). In conclusion, integrating all four complementary analytical modes allows comprehensive characterization of differential ion features, supporting the development of classifiers for geographical authentication.

### 3.2 Univariate threshold authentication of Zimbabwe and yunnan tobaccos by similarity scores between CIF matrices

NTA based on LC-HRMS inherently produces large and highly dimensional fingerprints of ion features. Developing a facile and definitive classification system for the routine authentication of Yunnan and Zimbabwe tobacco extracts requires simplifying this complex spectral data into a univariate format with strict threshold values. A highly effective approach involves evaluating the similarity between the holistic fingerprints of assessed samples and reference models, which is often determined through cosine or dot product (DP) calculations (Wang et al., 2023). Although this methodology successfully differentiates among distinct species, it encounters limitations when applied to numerous samples of the same species, such as tobacco extracts. This difficulty stems from the highly conserved genome sequences and metabolic pathway, which results in heavily overlapping chemical profiles. For instance, 94.1% of ion features show no significant differences when comparing Zimbabwe tobacco to other extracts. Similarly, 99.6% of ion features remain indistinguishable when comparing Yunnan to other varieties. This conserved biological background yields a vast majority of ubiquitous metabolites, which disproportionately inflate baseline similarity. As such, the distinct chemical signatures indicative of specific geographical origins would be largely obscured, making their isolation a significant analytical challenge. To address this limitation, here we refined the methodology to calculate the DP score of individual samples specifically against established Characteristic Ion Feature (CIF) matrix for each premium tobacco extract, thereby bypassing the use of holistic fingerprints. By restricting the comparison to the ion features defined within the Zimbabwe and Yunnan CIF matrices, non-discriminative background variance is systematically eliminated. Consequently, the derived similarity score accurately reflects the complex multidimensional profile of geographic markers, rather than coincidental overlaps of ubiquitous metabolites.

When evaluated against the Zimbabwe CIF matrix, the true Zimbabwe tobacco group exhibited DP values ranging from 0.0360 to 0.7265, with an mean score of 0.4623, whereas the non-Zimbabwe samples exhibit a substantially suppressed scores, averaging at 0.2218 (Supplementary Data Sheet 2; Supplementary Material). Conversely, the Yunnan CIF matrix displayed limited recognition power, owing to significant intra-group heterogeneity, resulting in marginal differentiation between true Yunnan samples (i.e., range: 0.0251–0.7306; mean: 0.2332) and non-Yunnan cohorts (i.e., range: 0.0075–0.6938; mean: 0.2254). Furthermore, a notable subset of non-premium samples exhibited unexpectedly high similarities. Specifically, 238 (15.5%) non-Zimbabwe samples and 498 (39.6%) non-Yunnan samples exhibited DP scores that exceeded the average values of their respective true sample groups. Although the general distribution of similarity scores indicates an underlying discriminative trend in targeted CIF matching, the substantial score overlaps between the premium tobacco group and the others necessitate the calibration of specific analytical thresholds. Implementing a rigorously selected cut-off value is therefore imperative to maximize true positive identification while mitigating false positive ratios.

To determine the optimal decision boundaries for these univariate scores, we evaluated two distinct mathematical algorithms: Probability Density Function (PDF) intersection and Youden’s J statistic. These methods were selected because they provide objective, mathematically grounded cut-offs without relying on arbitrary operational penalties (Nichani et al., 2023; Turkina et al., 2025). The PDF intersection serves as a theoretical baseline, assuming the similarity scores follow a Gaussian distribution and calculating the exact coordinate where the probability densities of the target and non-target classes are equal. Conversely, Youden’s J statistic serves as an empirical optimizer derived from the Receiver Operating Characteristic (ROC) curve. Independent of the underlying data distribution, it systematically scans the raw values to pinpoint the threshold that strictly maximizes the sum of the True Positive Rate (TPR) and True Negative Rate (TNR).

**FIGURE 2:**
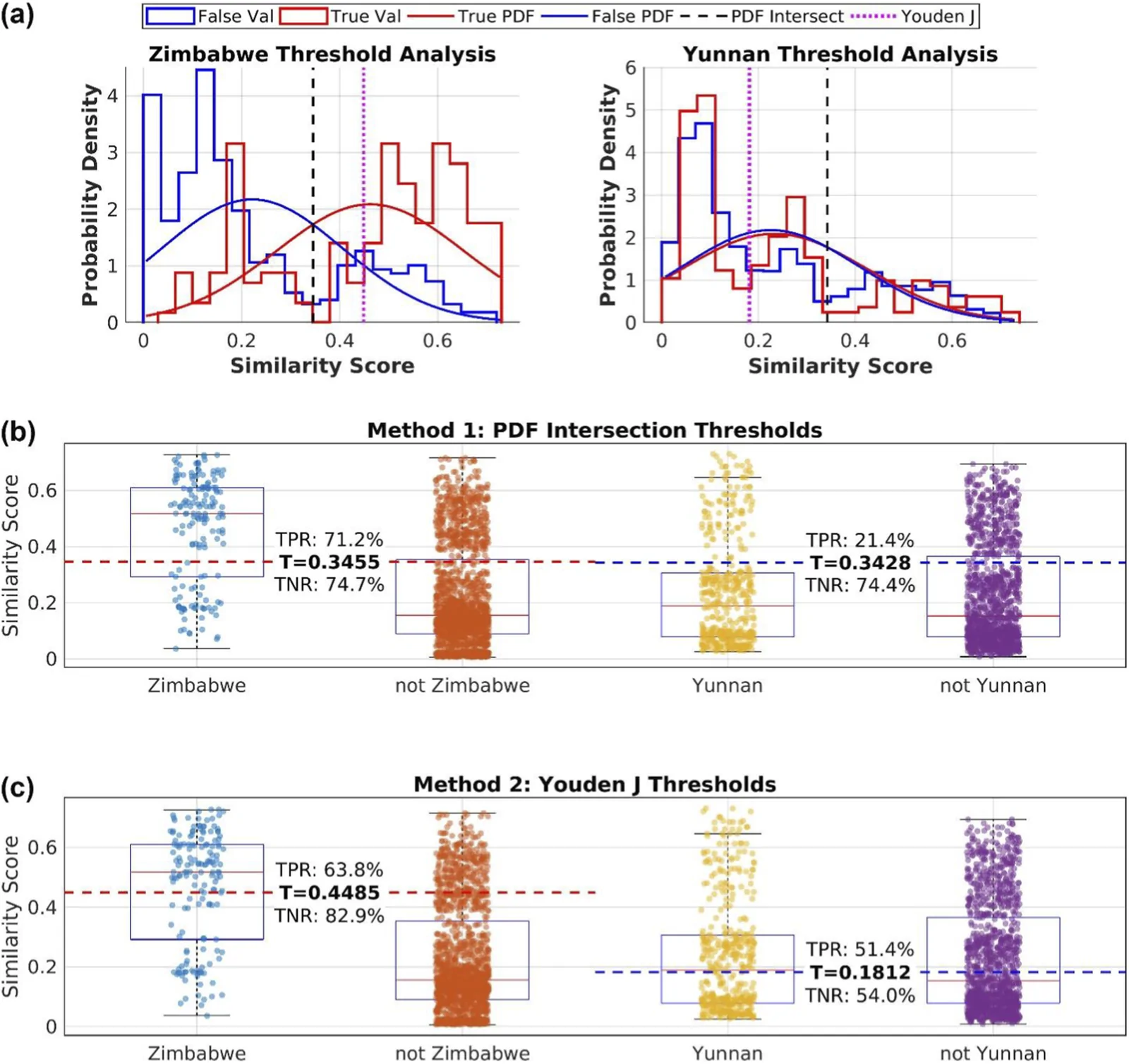
Dot product threshold optimization and classification performance of the Characteristic Ion Feature (CIF) matching methodology for geographical authentication of premium tobacco extracts. **(a)** Probability density distributions of dot product (DP) values for authentic (red) and non-authentic (blue) samples evaluated against the Zimbabwe CIF matrix (left) and the Yunnan CIF matrix (right). Step histograms represent the empirical data, while solid curves illustrate the theoretical Gaussian distributions derived from group means and standard variances. Vertical lines indicate decision boundaries established by the Probability Density Function (PDF) intersection (black dashed line) and Youden’s J statistic (magenta dotted line) **(b,c)** Authentication performance based on cut-offs determined by the PDF intersection and Youden’s J thresholds, respectively. Box plots are overlaid with jittered scatter points to visualize individual sample density. Each applied threshold (T) is marked by a horizontal dashed line alongside the resulting True Positive Rate (TPR) and True Negative Rate (TNR).

An evaluation of the authentication results for all the 1,697 tobacco extracts reveals distinct mathematical behaviors between the two algorithms (Figure 2). The probability density histograms visually confirm the discriminative capability of the methodology while highlighting the underlying biological complexity of the samples (Figure 2a). In both the Zimbabwe and Yunnan datasets, the authentic sample groups (red traces) form a broad distribution across the higher scoring ranges, while the non-authentic samples (blue traces) are densely concentrated at the lower end of the similarity scores. Notably, the probability density histograms reveal a distinct clustering of authentic samples within the lower similarity range of 0.1–0.2. Because this sub-population of authentic samples falls below the mathematically derived thresholds, it would lower the true positive rate (TPR) of the method. The fact that these erroneously classified false negatives form a concentrated cluster, rather than a uniform statistical spread, strongly suggests the existence of distinct sub-classes within the primary authentic cohort. This variance indicates that beyond the overarching geographical origin, additional unpredicted factors, such as differing microclimates, distinct agricultural practices, or variations in the extraction process, significantly alter their chemical signature. The PDF intersection method established DP thresholds (T) of 0.3455 for Zimbabwe and 0.3428 for Yunnan tobacco extracts. These boundaries resulted in a TPR of 71.2% and a TNR of 74.7% for Zimbabwe authentication, alongside a TPR of 21.4% and a TNR of 74.4% for the Yunnan cohort (Figure 2b). In comparison, Youden’s J method raised the Zimbabwe threshold to 0.4485 and lowered the Yunnan threshold to 0.1812. For Zimbabwe, this shift slightly reduced the TPR to 63.8% but increased the TNR to 82.9%, yielding minor gains in both balanced and overall accuracy. Conversely, the lower threshold for Yunnan authentication significantly boosted its TPR to 51.4% with relatively small reduction of TNR to 54.0% (Figure 2c).

While the DP score evaluates the spectral patterns of constituent markers, it remains independent of their relative concentration. Thereby, the utilization of DP scores defined CIF matrices natively enables an orthogonal score to determine the authenticity of a query sample based on the intensity ratio of its constituent markers. This univariate metric, termed as the R score, is defined as the summed intensity of all CIFs relative to the total intensity of all detectable ion features within the sample. Furthermore, because pattern similarity and relative concentration represent two independent mathematical properties, they were integrated into a combined score calculated as the product of DP and R scores. This combined metric functions as a stringent logical “AND” gate, requiring an extract to possess both the correct spectral shape and sufficient marker abundance to achieve a high score. Based on the DP scores, R scores, and the combined scores (Combo) of all the 1,697 samples, the performances of the univariate threshold authentication method are compared (Table 1, Supplementary Data Sheets 2–4, Supplementary Material).

**TABLE 1:** Performance metrics of different scoring methods and thresholding algorithms for Zimbabwe and Yunnan tobacco authentication.

Target	Scoring	Algorithm	Threshold	TPR (%)	TNR (%)	Balanced accuracy (%)	Overall accuracy (%)
Zimbabwe	DP	PDF	0.3455	71.2	74.7	73.0	74.4
		Youden J	0.4485	63.8	82.9	73.4	80.9
	R	PDF	0.3562	85.3	63.4	74.4	65.3
		Youden J	0.4472	73.0	82.7	77.9	81.8
	Combo	PDF	0.1692	67.5	80.2	73.9	79.0
		Youden J	0.0849	82.2	70.7	76.5	71.7
Yunnan	DP	PDF	0.3428	21.4	74.4	47.9	60.6
		Youden J	0.1812	51.4	54.0	52.7	53.3
	R	PDF	0.3617	23.4	79.7	51.6	65.1
		Youden J	0.1811	52.7	57.4	55.1	56.2
	Combo	PDF	0.1747	18.2	82.5	50.4	65.8
		Youden J	0.0273	53.4	55.3	54.4	54.8

A systematic comparison of the performance metrics reveals that the non-parametric Youden’s J method consistently outperforms the parametric PDF intersection method in terms of balanced accuracy. For the Zimbabwe cohort, the Youden’s J, R score achieved a higher balanced accuracy of 77.9%, whereas the PDF R score reached only 74.4%. Similarly, in the Yunnan cohort, the Youden’s J, R score produced a balanced accuracy of 55.1%, compared to 51.6% for the PDF method. This superiority stems from its ability to scan empirical data without assuming a specific distribution. Because the tobacco extract similarity scores exhibit non-Gaussian behavior with asymmetrical tails, the assumption of Gaussian distribution by PDF method often results in statistically biased thresholds. For instance, the Zimbabwe PDF R score threshold of 0.3562 resulted in a TNR of only 63.4%, whereas the Youden J threshold of 0.4472 successfully mitigated false positives to reach a TNR of 82.7%.

Among the three scoring systems, the R score emerged as the most reliable metric for both premium tobacco extracts. While the DP score captures spectral patterns, the R score accounts for the weight of discriminant markers relative to the holistic fingerprints. In the Zimbabwe cohort, the Youden’s J, R score (77.9% balanced accuracy) outperformed the Youden’s J, DP score (73.4% balanced accuracy). The combined score (Combo) functions as a stringent logical “AND” gate, but the result indicates it may be overly conservative. For the Yunnan cohort, the Combo PDF method reached a high TNR of 82.5%, yet its balanced accuracy was limited to 50.4%, because the dual requirement suppressed the TPR to 18.2%, the lowest in the group.

For the authentication of either Zimbabwe or Yunnan tobacco, the R score incorporated with Youden’s J threshold is the best authentication strategy. This specific configuration yielded the highest balanced accuracy for both groups, reaching 77.9% for Zimbabwe and 55.1% for Yunnan. However, it is critical to note that regardless of the thresholding algorithm selected, an inherent mathematical trade-off between TPR and TNR remains inevitable. This inverse relationship highlights the intrinsic limitation of univariate threshold specification methods. Consequently, a univariate threshold strategy is too rough to handle the nuanced chemical profiles of real-world tobacco extracts. Because the variable of geographic origin interacts with multiple unpredicted variables, authentic samples inevitably split into distinct sub-classes that a single scalar score cannot effectively separate. Addressing this multi-layered variation necessitates a multivariate and non-linear classification approach.

### 3.3 Machine learning models for the authentication of Zimbabwe and yunnan tobaccos trained by CIF matrices

While univariate thresholding methods achieve an overall accuracy of approximately 53%–82%, their specificity and precision are fundamentally constrained by the inherent complexity of fingerprints derived from NTA. Specifically, LC-HRMS NTA fingerprints of tobacco extracts exhibit extreme dimensionality, routinely encompassing tens of thousands of distinct ion features. Within these multidimensional spaces, chemical signals are highly susceptible to matrix effects and natural variations, which frequently confound traditional linear classification boundaries. Consequently, ML algorithms are optimally suited for multivariate classification systems because of their mathematical capacity to model nonlinear relationships. By simultaneously evaluating the complete high-dimensional feature space, these nonlinear models account for complex and orthogonal fingerprint characteristics, thereby establishing a statistically rigorous and highly accurate framework for the geographic authentication of premium Zimbabwe and Yunnan tobacco extracts.

**FIGURE 3:**
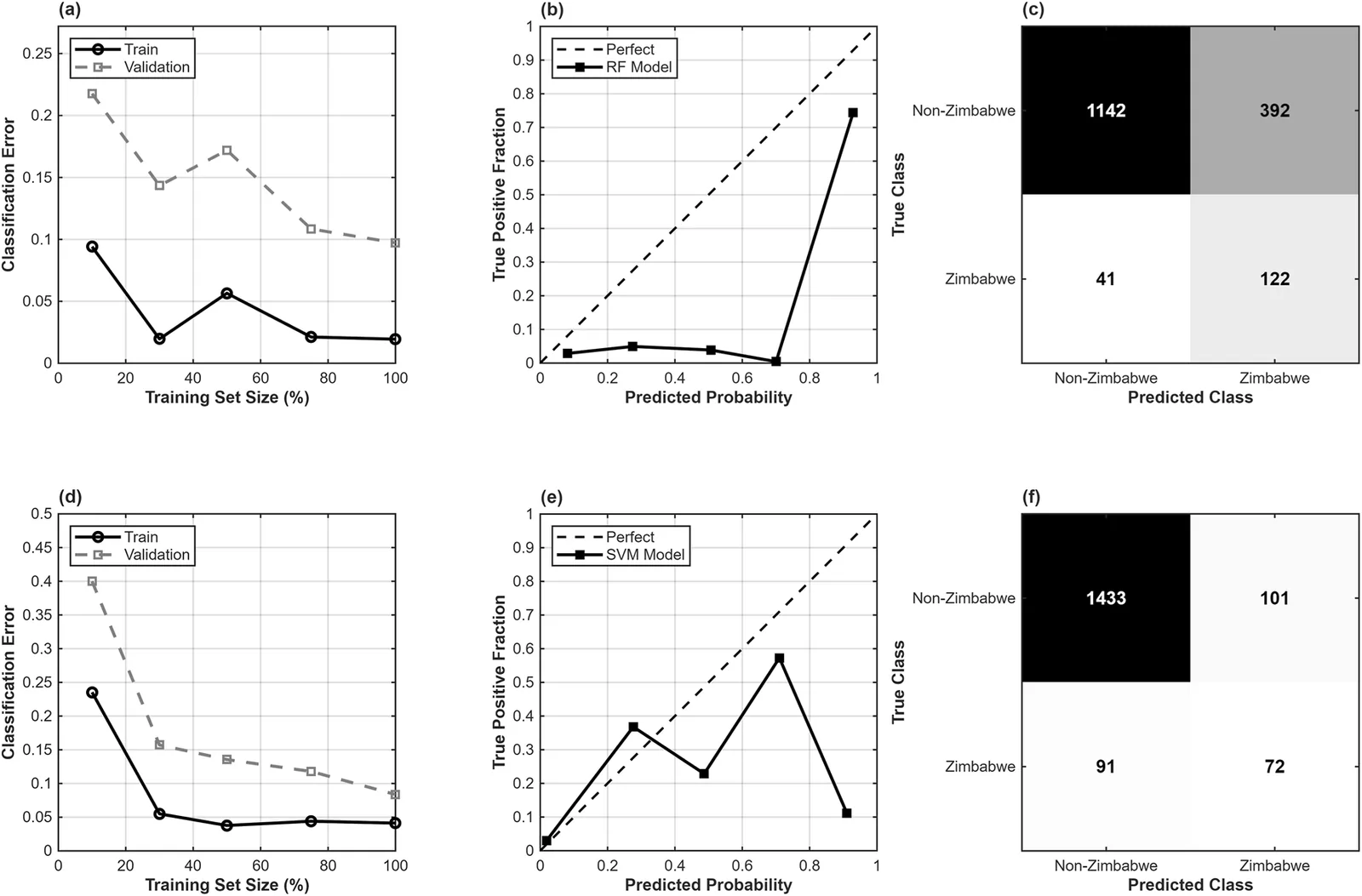
Performance and overfitting diagnostics for the Zimbabwe classification models. The top row illustrates the diagnostic evaluation for the RF-Zimbabwe model, and the bottom row displays the evaluation for the SVM-Zimbabwe model **(a,d)** Learning curves plotting classification error against training set size (%). The stable convergence between the training error (solid black lines) and cross-validation error (dashed gray lines) as data size increases demonstrates adequate model capacity and minimal overfitting **(b,e)** Calibration plots evaluating the reliability of the models’ predicted probabilities compared to the observed true positive fractions. The dashed diagonal line represents perfect calibration; note the alignment achieved in the SVM model via Platt scaling **(c,f)** Leave-one-batch-out (LOBO) confusion matrices detailing the final predictive accuracy for distinguishing Non-Zimbabwe and Zimbabwe instances.

To ensure the validity and generalizability of our computational pipeline, we conducted a comprehensive evaluation of model hyperparameter optimization, class imbalance adjustments, and overfitting diagnostics. To overcome the curse of dimensionality, we restricted the initial candidate pool to 1,000 features, ultimately retaining between 803 and 974 features across cohorts (i.e., 891 for RF-Zimbabwe; 974 for RF-Yunnan; 889 for SVM-Zimbabwe; 803 for SVM-Yunnan) to maximize signal while minimizing redundant noise. Model optimization yielded distinct hyperparameter configurations: RF architectures required 184 trees (min leaf size: 4) for Zimbabwe and 182 trees (min leaf size: 4) for Yunnan, while SVM models optimized to a Box Constraint (*C*) of 230.06 with a Kernel Scale (γ^−1^) of 62.26 for Zimbabwe, and *C* 713.71 with γ^−1^ 18.78 for Yunnan. Cost-sensitive matrices effectively penalized minority-class misclassifications (i.e., penalties ranging from 4.37 to 28.20), and Platt scaling successfully calibrated posterior probability outputs for the SVM classifiers (Table 2). Evaluation of learning curves demonstrated parallel convergence between training and validation metrics without high-variance divergence, indicating minimal overfitting (Figure 3). Permutation testing against randomized label distributions further confirmed that the predictive capacity of all models was statistically robust, consistently achieving significance (p = 0.0196).

**TABLE 2:** Model Configuration and hyperparameters of different machine learning (ML) models for Zimbabwe and Yunnan tobacco authentication.

Parameter	RF-Zimbabwe	RF-yunnan	SVM-Zimbabwe	SVM-yunnan
Selected features (max 1,000)	891	974	889	803
Minority class cost penalty	25.00	4.37	28.20	6.26
Probability threshold	Default	Default	Platt scaling	Platt scaling
Number of trees	184	182	N/A	N/A
Minimum leaf size	4	4	N/A	N/A
Box constraint (C)	N/A	N/A	230.06	713.71
Kernel scale (γ^−1^)	N/A	N/A	62.26	18.78

Despite all models performing significantly better than chance, the final discriminative metrics highlight stark regional disparities in practical efficacy. RF-Zimbabwe achieved superior overall performance with a Balanced Accuracy of 74.65% (95% CI: 71.09%–77.97%), an Area Under the Receiver Operating Characteristic Curve (ROC AUC) of 0.8445, and an Area Under the Precision–Recall Curve (PR AUC) of 0.7405, while SVM-Zimbabwe reached an overall accuracy of 88.69% (95% CI: 87.09%–90.22%) and ROC AUC of 0.7977. Conversely, while statistically valid, both Yunnan models suffered from compromised sensitivity (20.68% for RF; 22.05% for SVM) and low ROC AUCs (0.4741 for RF; 0.4836 for SVM) and PR AUCs (0.3518 for RF; 0.2766 for SVM). This divergence underscores that while the algorithms successfully identified underlying patterns in both datasets, isolating the positive class in the Yunnan cohort presents a significantly greater computational challenge, underscoring the necessity of cohort-specific modeling strategies (Table 3; Figure 3).

**TABLE 3:** Performance metrics of different machine learning (ML) models for Zimbabwe and Yunnan tobacco authentication.

Metric	RF-Zimbabwe	RF-yunnan	SVM-Zimbabwe	SVM-yunnan
Overall accuracy (95% CI)	74.48% (72.42–76.37)	73.66% (71.54–75.81)	88.69% (87.09–90.22)	70.83% (68.53–73.10)
Sensitivity (95% CI)	74.85% (68.09–81.49)	20.68% (16.74–24.40)	44.17% (36.26–52.47)	22.05% (18.20–25.95)
Specificity (95% CI)	74.45% (72.37–76.54)	92.20% (90.65–93.63)	93.42% (92.09–94.78)	87.91% (86.08–89.71)
Balanced accuracy (95% CI)	74.65% (71.09–77.97)	56.44% (54.38–58.38)	68.79% (64.72–73.01)	54.98% (52.79–57.12)
ROC AUC	0.8445	0.4741	0.7977	0.4836
PR AUC	0.7405	0.3518	0.3286	0.2766
Permutation test p value	0.0196	0.0196	0.0196	0.0196

The superior overall performance of the RF ensemble over the RBF-SVM, most notably in achieving a balanced classification for the Zimbabwe cohort (Balanced Accuracy: 74.65% vs. 68.79%; Sensitivity: 74.85% vs. 44.17%), stems from fundamental architectural advantages in handling nontargeted metabolomic data. LC-HRMS fingerprints inherently exhibit severe multicollinearity, as co-eluting adducts, isotopes, and fragment ions generate clusters of highly correlated variables. Based on PCA importance measures for feature selection, we successfully filtered the initial feature pool to prioritize variables driving maximum variance. However, the differential performance between RF-Zimbabwe and SVM-Zimbabwe models indicate that residual collinearity may still persist among selected components. While continuous kernel methods like RBF-SVM can remain sensitive to local collinear noise when constructing decision hyperplanes in high-dimensional spaces (∼800–900 features), RF naturally mitigates multicollinearity through randomized feature subspace partitioning at each decision node. Furthermore, RF ensembles demonstrate exceptional resistance to overfitting by averaging multiple decorrelated decision trees, effectively suppressing variance without inflating bias. The step-wise axis-aligned decision rules of decision trees are also inherently better equipped to model nonlinear threshold-based chemical abundances than the smooth geometric boundaries of RBF kernels.

Chemical annotation of the 891 CIFs selected in the RF-Zimbabwe model yielded 148 putative identifications, providing crucial biological context to the statistical discrimination of Zimbabwe tobacco extracts (Supplementary Data Sheet 5, Supplementary Material). The annotated features predominantly encompass key plant secondary metabolite classes known to be highly responsive to terroir, including phenolic compounds, terpenoids, and complex lipids (Wu et al., 2025). Notably, the model heavily weights phenolic acids and their derivatives, such as rosmarinic acid and 3,5-dicaffeoylquinic acid. They are well-documented stress-response metabolites in Nicotiana tabacum known to fluctuate significantly based on soil composition, UV exposure, and curing practices (Martin et al., 2023). Additionally, the presence of distinct terpenoids (e.g., fusaridioic acid A, vachanic acid, and 7-ketodehydro-abietic acid) and glycosides (e.g., kanokoside A, poliumoside, and galactinol) highlights the role of geographical origin in shaping the defense and flavor-precursor profiles of plants (De Rosso et al., 2022). The inclusion of various structural lipids (e.g., MGDG, LPE, and PC species) further suggests that environmental factors unique to Zimbabwe influence the foundational membrane lipid remodeling of the tobacco plants (Murphy et al., 2025). Ultimately, interpreting these discriminant CIFs confirms that the authentication power of RF-Zimbabwe model is not merely a product of random statistical variance, but is deeply rooted in the chemically distinct and biologically meaningful metabolic signatures imparted by the specific agroecological conditions of the region.

While these 148 annotated features provide essential biological context, it is equally important to recognize that the majority of the 891 selected CIFs remain unannotated. This large fraction of unidentified signals exemplifies the vast reservoir of chemical information frequently detected by high-resolution omics technologies but rarely characterized. Rather than excluding these unknowns, our methodology intentionally leverages this unannotated chemical space for predictive modeling. By doing so, this study highlights a novel strategy in nontargeted metabolomics and Foodomics, showing that the global metabolome, including its chemical dark matter, enables robust food authentication without the need for comprehensive metabolite identification.

### 3.4 Validation with lab-made tobacco extracts

The trained RF model exhibited good discriminatory capability when deployed on the validation set, successfully achieving accurate classification across most tested samples (Table 4). The freshly prepared, unadulterated positive controls (authentic Zimbabwe extracts) were accurately predicted as “Authentic”, yielding confidence probabilities between 57.1% and 61.3%. Notably, extracts subjected to a 2-week storage period at room temperature attained relatively lower probability scores compared to their freshly prepared counterparts. Although Zimbabwe Extract 2 (Series 3) was misclassified as “Fake/Other”, RF-Zimbabwe model assigned its probability at 48.6%, significantly higher than the scores observed for the truly adulterated extracts.

**TABLE 4:** Validation results of the random forest model for Zimbabwe tobacco extract authentication.

Series	Sample description	Model prediction	Probability (authentic)
Series 1	Zimbabwe extract 1	Authentic	61.3
	Zimbabwe extract 2	Authentic	57.1
Series 2	Zimbabwe extract 1 (stored 2 weeks)	Authentic	54.6
	Doped in non-Zimbabwe tobacco (1,000 ppm)	Fake/Other	22.9
	Doped in non-Zimbabwe tobacco (100 ppm)	Fake/Other	17.5
	Doped in non-Zimbabwe tobacco (10 ppm)	Fake/Other	24.8
	Non-Zimbabwe tobacco	Fake/Other	20.2
Series 3	Zimbabwe extract 2 (stored 2 weeks)	Fake/Other	48.6
	Doped in non-Zimbabwe tobacco (1,000 ppm)	Fake/Other	22.3
	Doped in non-Zimbabwe tobacco (100 ppm)	Fake/Other	19.2
	Doped in non-Zimbabwe tobacco (10 ppm)	Fake/Other	19.2
	Non-Zimbabwe tobacco	Fake/Other	14.3

Crucially, the RF model exhibited high sensitivity against adulteration. Across both Series 2 and 3, all heavily diluted samples representing fake or low-quality market products (0–1,000 ppm authentic content) were successfully classified as “Fake/Other”. The probability of authenticity for these adulterated samples plummeted to a narrow margin of 14.3%–24.8%. This sharp contrast in probability scores between the genuine and adulterated samples confirms that the RF model is not easily bypassed by the presence of trace amounts (up to 1,000 ppm) of authentic Zimbabwe extract. These validation results confirm that the RF model is highly effective at detecting economically motivated adulteration, though its robustness regarding sample aging and storage conditions requires further refinement.

## 4 Conclusion

This study successfully established a machine learning-driven analytical framework for the geographic authentication of premium Zimbabwe and Yunnan tobacco extracts. Integrating liquid chromatography-high resolution mass spectrometry with rigorous statistical filtering allowed for the construction of Characteristic Ion Feature matrices, effectively transforming vast, unannotated chemical data into predicative variables. This approach emphasizes the ability of machine learning to utilize both annotated and unannotated metabolomic information for authentication purposes, demonstrating that holistic fingerprinting can deliver robust predictive models without relying on comprehensive structural identification. While multivariate models successfully navigated high-dimensional spectral data to accurately authenticate and detect adulteration in the Zimbabwe cohort, both univariate and multivariate methods failed to provide reliable authentication for the Yunnan extracts. This limitation stemmed from immense intra-group heterogeneity caused by diverse supply networks and varying processing treatments, which heavily diluted the shared chemical markers and hindered the isolation of a singular, homogenous fingerprint. Ultimately, this methodology provides a highly adaptable template for the authentication of stable agricultural commodities, while highlighting the necessity of developing more granular, cohort-specific modeling strategies for highly complex and diverse matrices. Despite its successful applications on the Zimbabwe cohort, this framework remains subject to certain limitations. The initial training models rely heavily on the label reliability of market-sourced samples, and the potential impact of long-term storage or processing batch effects requires broader external validation to ensure marker stability over time. Finally, while the inclusion of unannotated features maximizes classification power, the lack of definitive feature annotation limits the complete biological interpretation of the specific metabolic pathways driving these regional differences.

## Data Availability

The original contributions presented in the study are included in the article/Supplementary Material, further inquiries can be directed to the corresponding authors.
